# Down-Regulation of 11β-Hydroxysteroid Dehydrogenase Type 2 by Bortezomib Sensitizes Jurkat Leukemia T Cells against Glucocorticoid-Induced Apoptosis

**DOI:** 10.1371/journal.pone.0067067

**Published:** 2013-06-24

**Authors:** Yi Tao, Lu Gao, Xiaosong Wu, Hongmei Wang, Guang Yang, Fenghuang Zhan, Jumei Shi

**Affiliations:** 1 Department of Hematology, Shanghai Tenth People’s Hospital, Tongji University School of Medicine, Shanghai, People’s Republic of China; 2 Department of Physiology, Second Military Medical University, Shanghai, People’s Republic of China; 3 Department of Internal Medicine, University of Iowa Carver College of Medicine, Iowa City, Iowa, United States of America; Emory University, United States of America

## Abstract

11β-hydroxysteroid dehydrogenases type 2 (11β-HSD2), a key regulator for pre-receptor metabolism of glucocorticoids (GCs) by converting active GC, cortisol, to inactive cortisone, has been shown to be present in a variety of tumors. But its expression and roles have rarely been discussed in hematological malignancies. Proteasome inhibitor bortezomib has been shown to not only possess antitumor effects but also potentiate the activity of other chemotherapeutics. In this study, we demonstrated that 11β-HSD2 was highly expressed in two GC-resistant T-cell leukemic cell lines Jurkat and Molt4. In contrast, no 11β-HSD2 expression was found in two GC-sensitive non-hodgkin lymphoma cell lines Daudi and Raji as well as normal peripheral blood T cells. Inhibition of 11β-HSD2 by 11β-HSD inhibitor 18β-glycyrrhetinic acid or 11β-HSD2 shRNA significantly increased cortisol-induced apoptosis in Jurkat cells. Additionally, pretreatment of Jurkat cells with low-dose bortezomib resulted in increased cellular sensitivity to GC as shown by elevated induction of apoptosis, more cells arrested at G1 stage and up-regulation of GC-induced leucine zipper which is an important mediator of GC action. Furthermore, we clarified that bortezomib could dose-dependently inhibit 11β-HSD2 messenger RNA and protein levels as well as activity (cortisol-cortisone conversion) through p38 mitogen-activated protein kinase signaling pathway. Therefore, we suggest 11β-HSD2 is, at least partially if not all, responsible for impaired GC suppression in Jurkat cells and also indicate a novel mechanism by which proteasome inhibitor bortezomib may influence GC action.

## Introduction

Glucocorticoids (GCs), stress hormones secreted from the adrenal gland, are physiologically involved in metabolism, cell differentiation, and several aspects of the maintenance of homeostasis. They play their parts by combining with cognate intracellular glucocorticoid receptor (GR) and translocating to the nucleus afterwards [1]. Pharmacologically, GCs have pro-apoptotic effects and are administered for the treatment of lymphoproliferative disorders [2]. In childhood acute lymphoblastic leukaemia (ALL) treatment protocols, an introductory mono-therapy GC has been used to reduce leukemic blasts in GC sensitive patients in the initial therapy. However, GC sensitivity is different from person to person and GC-resistance is a therapeutic problem with an unclear molecular mechanism. Some studies have suggested that the GC receptor is underexpressed or mutated in GC-resistant cells, but others have reached contradictory results [3], [4], [5], indicating the possibility of multiple diverse mechanisms involved in GC resistance.

GC concentrations in target cells depend not only on their extracellular concentrations, but additionally on an intracellular prereceptor control mechanism constituted by 11β-hydroxysteroid dehydrogenase (11β-HSD) enzymes. 11β-HSD1 activates GCs (from inactive 11-keto forms cortisone to cortisol), whereas 11β-HSD2 inactivates GCs by exclusively converting active cortisol to inactive cortisone [6]. These two enzymes represent pre-receptor mechanism controlling the ratio of the local concentrations of biologically active GCs. It is noteworthy that ectopic expression 11β-HSD2 has been described in a number of solid tumors including breast cancer, colon carcinoma and pituitary adenoma [7], [8], [9]. Specifically, some researchers have described a shift from predominant 11β-HSD1 expression in normal tissue to 11β-HSD2 in tumors [10]. Of importance, Nigawara et al. reported that abnormally expressed 11β-HSD2 resulted in the decreased GC suppression in corticotroph adenoma [11]. However, until now, the expression of 11β-HSD2 and its association with GC resistance have rarely been discussed in hematological malignancies, such as lymphoblastic leukemia and lymphoma.

Bortezomib (Velcade, PS-341) is the first proteasome inhibitor that was clinically tested in patients and becomes a therapeutic modality for multiple myeloma [12]. Moreover, bortezomib is also highly cytotoxic to a variety of malignancies. The antitumor mechanism of bortezomib not only promotes apoptosis in cancer cells, but also sensitizes these cells to chemotherapy [13]. In addition, bortezomib has been demonstrated to overcome GC resistance at the hypoxic blood-brain barrier to reduce brain edema in acute ischemic stroke [14]. But it is unclear if bortezomib could increase cell susceptibility to GC-induced cytotoxicity.

In this study, we investigated the 11β-HSD2 expression in GC-resistant T-cell lymphoblastic lymphoma/leukemia lines and further determined its contribution to GC resistance by using 11β-HSD inhibitor or 11β-HSD2 gene silencing. To clarify whether bortezomib could improve GC sensitivity, we treated Jurkat T-cell lymphoblastic lymphoma/leukemia cells with cortisol following bortezomib pretreatment. Herein we reported 11β-HSD2 presence was partly responsible for GC resistance in leukemia T cells and bortezomib enhanced GC sensitivity in Jurkat cells by P38 mitogen-activated protein kinase (MAPK)-mediated down-regulation of 11β-HSD2, suggesting that 11β-HSD2 could be used as a potential therapeutic target in GC-resistant lymphoproliferative disorders and also a novel downstream target of bortezomib action.

## Materials and Methods

### Cell Lines and Cell Treatment

Jurkat and Molt4, two T-ALL cell lines, were kindly provided by Dr. J. Hou (Second Military Medical University, Shanghai, China). Daudi and Raji, two Non-hodgkin lymphoma (NHL) cell lines, were derived from ATCC (Manassas, VA, USA). Cells were grown in RPMI 1640 (Bio Whitaker, Rockland, ME, USA) at 37°C, 5% carbon-dioxide and saturated humidity. The media were supplemented with 10% fetal calf serum (Sigma), 100 U/ml penicillin, 100 mg/ml streptomycin and 2 mM L-glutamine (GibcoBRL, Paisley, UK). Peripheral blood samples were obtained from healthy volunteers after written informed consent in accordance with Declaration of Helsinki. Approval was obtained by Shanghai Tenth People’s Hospital for Medical Sciences Institutional Review Board for these studies. T cells were isolated from peripheral blood using CD3 positive magnetic bead selection (Stem Cell Technologies). Jurkat cells in log-phase growth were treated with increasing concentrations of bortezomib (Millennium Pharmaceuticals, USA) for 12 or 24 hours before the detection of 11β-HSD2 mRNA and protein expression. After 24 hours of indicated concentrations of bortezomib exposure, Jurkat cells were collected and washed once with medium and treated with indicated concentrations of cortisol (Sigma) for 48 hours. Cells unexposed to bortezomib were used as a control. For analysis of 11β-HSD2 inhibition, Jurkat cells were treated with 5 µM 11β-HSD inhibitor 18β-glycyrrhetinic acid (Sigma) and 10 µg/ml cortisol in the presence or absence of GC receptor antagonist RU486 (1 µM) for 48 hours. For analysis of p38 MAPK signal pathway, Jurkat cells were pretreated with 5 nM bortezomib in the presence or absence of 10 µM p38 MAPK inhibitor SB203580 (Cell Signaling Technology, MA, USA) for 24 hours. Then cells were exposed to 10 µg/ml cortisol for 48 hours.

### Reverse Transcriptase–polymerase Chain Reaction (RT-PCR) and Quantitative Real-time PCR (qRT-PCR)

Total RNA was prepared from individual samples by using TRIzol reagent (Invitrogen, USA), and 5 µg total RNA was used for the RT reaction using the PrimeScript™ RT reagent kit (Takara, Tokyo, Japan) and then stored at −20°C. The cDNA samples obtained was then amplified by PCR with Taq DNA polymerase (Takara, Tokyo, Japan). The PCR condition used was as follows: 96°C, 3 min, for initial denaturation; 95°C (15 sec), 64°C (15 sec) and 72°C (15 sec) in a total 40 cycles with a final extension step at 72°C for 10 min. The nucleotide sequences of the primers were as follows: 11β-HSD2, sense: 5′-GCTGCTGCGCTCAGACCT-3′, antisense: 5′-GGCTGTTCAACTCCAATACG-3′ (accession number NM_000196). β-actin, sense: 5′-TGTGTTGGCGTACAGGTCTTTG-3′, antisense: 5′-GGGAAATCGTGCGTGACATTAAG-3′ (accession number NM_001101). All primer sets were designed to span at least one intron to avoid false-positive signals derived from genomic DNA. 10 µl of the reaction mixture were subsequently electrophoresed on a 1.5% agarose gel and visualized by ethidium bromide, using a 100 bp DNA ladder (Invitrogen) to estimate the band sizes. Placenta cDNA (from our own laboratory) was used as a positive control for 11β-HSD2.

To measure 11β-HSD2 and Glucocorticoid receptor α (GRα) mRNA levels, quantitative real-time PCR (qRT-PCR) was carried out using Rotor-Gene 3000 (Corbett Research, Sydney, Australia) in a total of volume of 25 µl reaction mixture following the manufacturer’s protocol using the 2×Taq PCR master mix (QIAGEN) and 0.2 µM of each paired primer. qRT-PCR conditions were optimized according to preliminary experiments to achieve linear relationships between initial RNA concentration and PCR product. The annealing temperature was set at 64°C and amplification cycles were set at 40 cycles. The nucleotide sequences of the primers were as follows: 11β-HSD2, sense: 5′-GTGCCATCGAGCTGCGTACCT-3′, antisense: 5′-TCCGCATCAGCAACTACTTCA-3′ (accession number NM_000196). GRα, sense: 5′-AACTGGCAGCGGTTTTAT-3′, antisens: 5′-ATTTTGGTATCTGATTGGTGAT-3′ (accession number NM_000176). GILZ, sense: 5′-AGCGTGGTGGCCATAGACAAC-3′, antisense: 5′-AGCCACTTACACCGCAGAA-3′ (accession number NM_198057). The specificity of the primers was verified by examining the melting curve as well as subsequent sequencing of the qRT-PCR products. Each sample was normalized on the basis of its β-actin mRNA content. The relative expression of the genes of interest was determined by using comparative threshold cycle (Ct) method. Briefly, ΔCt in each group was yielded by subtracting the Ct of the β-actin gene from the Ct of the target gene yields the ΔCt in each group (control and experimental groups). Then subtracting ΔCt of control group from the experimental group obtains the ΔΔCt, which was entered into the equation 2 ^- ΔΔCt^ and calculated for the exponential amplification of PCR.

### Western Blot Analysis

Cells were harvested and lysed with RIPA buffer (50 mM HEPES (pH 7.4), 150 mM NaCl, 1% Triton X-100, 30 mM sodium pyrophosphate, 5 mM EDTA, 2 mM Na_3_VO_4_, 5 mM NaF, 1 mM phenylmethyl-sulfonyl-fluoride (PMSF) and protease inhibitor cocktail). Protein lysates concentrations were measured using BCA assay (Pierce, Rockford, IL, USA). 50 µg of total cell lysates was used for SDS-PAGE. Western blot analyses of 11β-HSD2 (Santa Cruz Biotechnology, CA, USA), PARP, Caspase-3, Caspase-8, Caspase-9, phospho-p38 MAPK, p38 MAPK (Cell Signaling Technology, MA, USA) and β-actin (Sigma) were done on total cell lysates using specific monoclonal or polyclonal antibodies. Blots were washed with 1×PBS with Tween (PBST) and then incubated in IRDye 680 goat anti-mouse or IRDye 800 goat anti-rabbit secondary antibodies (LI-COR) for 1 hour, washed 3 times in 1×PBST, and scanned with an Odyssey Infrared Imaging System (LI-COR), as previously described [15]. The expression levels of β-actin were used as the loading control for the immunoblots.

### Specific 11β-HSD2 Gene Silencing by Lentivirus Expression Vector System

Two synthetic double-stranded oligonucleotide sequences specific for 11β-HSD2 silencing were purchased from Sigma. A nonsense scrambled oligonucleotide was used as a control. 11β-HSD2 short hairpin RNA (shRNA) double-stranded oligonucleotides were cloned into lentiviral pGMLV-SC1 vectors. Recombinant lentiviruses were produced by transient transfection of 293T cells. Efficiency of viral transfection was determined by counting the number of green fluorescent protein (GFP)–expressing cells using flow cytometry, and 95% transduction efficiency of Jurkat cells was achieved.

### 11β-HSD2 Activity Assay

11β-HSD2 oxidase activity was assayed using thin-layer chromatography (TLC)/liquid scintillation (LS). Briefly, cells were cultured in 12-well plates in the presence or absence of indicated concentrations of bortezomib for 24 hours, washed with PBS three times after the treatments. Then the cells were incubated with 1 µM cortisol (Sigma) containing 200,000 cpm [^3^H]cortisol (Amersham Life Science, Little Chalfont, UK) in FCS-free medium for 12 h at 37°C. Cells were then removed from the medium by centrifugation (10,000×g, 20 min, 4°C) and cell numbers per well were counted. To measure conversion, a mixture of cortisol and cortisone (40 µg each) was added to the collected medium to allow visual localization of the steroids when subjected to TLC. Steroids in the media were extracted with ethyl acetate. The extract was dried, reconstituted with ethyl acetate (100 µl), and applied to a TLC plate (Fisher Scientific, Pittsburgh, PA). Cortisol and cortisone were separated in the solvent system chloroform/ethanol (95∶5, vol/vol), steroids were visualized under UV light, scraped off, and extracted with ethyl acetate. The solvent was dried, scintillation fluid added, and the radioactivity counted in a liquid scintillation counter. 11β-HSD2 activity was expressed as a percentage: pmol cortisone produced per 10^6^ cells of the experimental sample/pmol cortisone produced per 10^6^ cells of the control sample.

### Cell Viability, Apoptosis and Cell Cycle Analyses

The live cells were counted by trypan blue dye exclusion assay for 5 days. Cell viability is expressed as the percent relative to control cells. For analysis of induction of apoptosis, cells were stained with Annexin V/PI (BD Pharmingen) according to the product instructions. Cells were then analyzed for apoptosis by flow cytometry using the Cell Quest software. For analysis of cell cycle, cells were washed with PBS, fixed in 70% ethanol overnight at −20°C, treated with RNaseA (Sigma, USA), stained with propidium iodide (Sigma), and assayed for DNA content by flow cytometry. The acquired histograms were analyzed by ModFit LT software to determine the cell cycle phase distribution.

### Statistical Analysis

All data are reported as mean ± SD. Paired Student’s t test or two-way ANOVA followed by the Student-Newman-Keuls test was used to assess significant differences between groups. Significance was set at p<0.05. The values for n refer to the number of experiments performed with different cell preparation.

## Results

### Jurkat and Molt4 Cells show Resistance to Cortisol-induced Apoptosis and Antiproliferation

**Figure 1:**
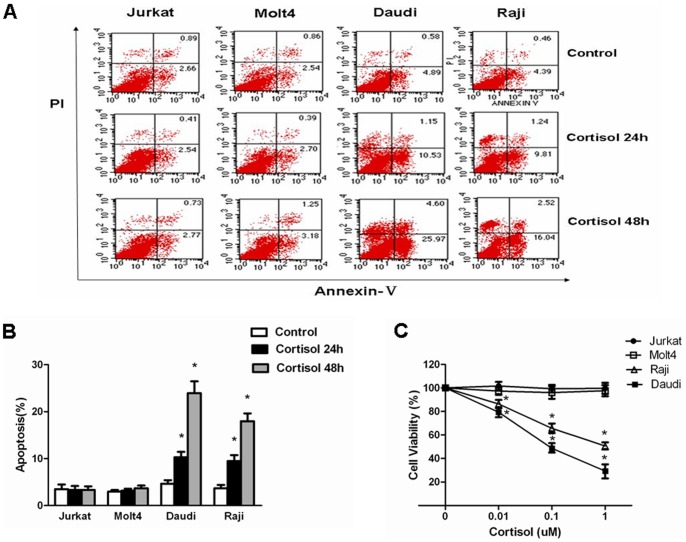
Resistance to cortisol-induced apoptosis and antiproliferation in Jurkat and Molt4 cells compared to Daudi and Raji cells. (A) Jurkat, Molt4, Daudi and Raji cells treated with 100 nM cortisol for 24 hours (middle panels) or 48 hours (bottom panels) were stained with Annexin V/PI and analyzed by flow cytometry. Annexin V single-positive cells were considered apoptotic. (B) Graphical representation of the data displayed in panel A as mean±SD of 3 independent experiments. *p<0.05 *vs.* untreated cells. (C) Jurkat, Molt4, Daudi and Raji cells treated with indicated concentrations of cortisol for 5 days. Percentages of viable cells after treatment were determined by using trypan blue dye exclusion. Data are shown as mean±SD of triplicate samples. *p<0.05 *vs.* untreated cells.

Firstly, we tested the cytotoxic effect of cortisol on Jurkat and Molt4 cells (two T-cell lymphoblastic lymphoma/leukemia cell lines) as well as Daudi and Raji cells (two non-hodgkin lymphoma cell lines). For this, we incubated each cell line with 100 nM cortisol for 24 and 48 hours and measured drug-induced apoptosis by Annexin V/PI staining. We observed that the drug was potent in inducing apoptosis in Daudi and Raji cells by 24 and 48 hours. In contrast, Jurkat and Molt4 cells showed resistance to cortisol-induced apoptosis (Fig. 1A and B).

We then proceeded to study the ability of cortisol to inhibit proliferation of each cell line in vitro. When the same cell lines were incubated with indicated concentrations of drug for 5 days, we observed a clear dose-dependent inhibition of proliferation of in Daudi and Raji cells tested (Fig. 1C). The inhibitory effect of cortisol on proliferation was evident even at a lower dose than was observed in the apoptosis assay. On the other hand, the drug imposed few effects on proliferation of Jurkat and Molt4 cells, a reflection of cell insensitivity to GC (Fig. 1C).

### High Expression of 11β-HSD2 and Failure in Auto-induction of GRα in Cortisol-resistant Cells

**Figure 2:**
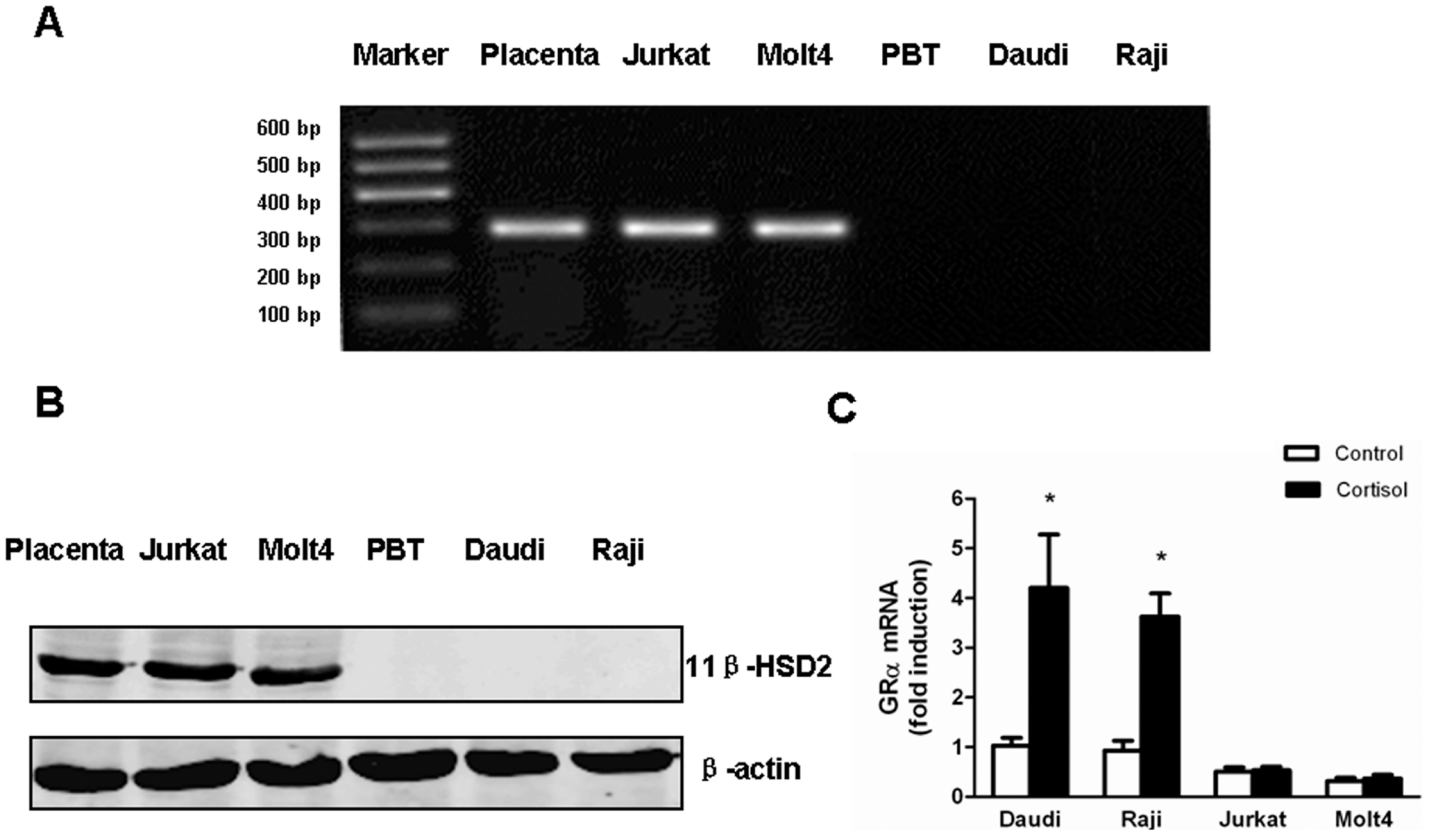
Jurkat and Molt4 cells express high expression of 11β-HSD2 and fail to auto-induce GRα after cortisol treatment. (A) 11β-HSD2 mRNA expression of each sample was detected using RT-PCR. These samples include (from left to right): Placenta, a 11β-HSD2 positive control; Jurkat, a T-ALL cell line; Molt4, a T-ALL cell line; PBT, peripheral blood T cells from healthy volunteers; Daudi, a B-NHL cell line; Raji, a B-NHL cell line. (B) Representative western blots showed 11β-HSD2 protein expression in each sample. 50 µg protein extracts from each sample were probed with 11β-HSD2 antibody. The expression levels of β-actin in the lysates served as the loading control. An immunoreactive protein of ∼42 kDa was found in Jurkat and Molt4 cells, but not in PBT, Daudi and Raji cells. (C) Jurkat, Molt4, Daudi and Raji cells were treated with 1 µM cortisol for 24 hours. GRα levels of cortisol-treated cells relative to those of untreated cells (control) in each sample were analyzed by quantitative real-time PCR. Data are shown as mean±SD of triplicate samples. *p<0.05 *vs.* untreated cells. There was no induction of GRα after cortisol treatment in Jurkat and Molt4 cells in comparison with Daudi and Raji cells.

The relative resistance to cortisol suggested the likely presence of 11β-HSD2 in Jurkat and Molt4 cells. Taking placental cells as a positive control, RT-PCR showed high levels of 11β-HSD2 mRNA expression in Jurkat and Molt4 cells. Whereas, 11β-HSD2 mRNA was not detectable in Daudi and Raji cells as well as peripheral blood T (PBT) cells from healthy volunteers (Fig. 2A). Consistent with 11β-HSD2 mRNA levels, western blotting showed high levels of 11β-HSD2 protein in Jurkat and Molt4 cells, but no detections in Daudi, Raji and normal PBT cells (Fig. 2B).

Due to a close relationship between GC sensitivity and GRα expression, qRT-PCR was applied to detect GRα level before and after cortisol treatment. The results showed that the basal level of GRα mRNA was higher in Daudi and Raji cells than Jurkat and Molt4 cells. In Daudi and Raji cells, GRα mRNA was significantly increased by cortisol treatment, whereas almost no induction of GRα occurred in Jurkat and Molt4 cells (Fig. 2C).

### Inhibition of 11β-HSD2 Increases Sensitivity to Cortisol-induced Apoptosis

**Figure 3:**
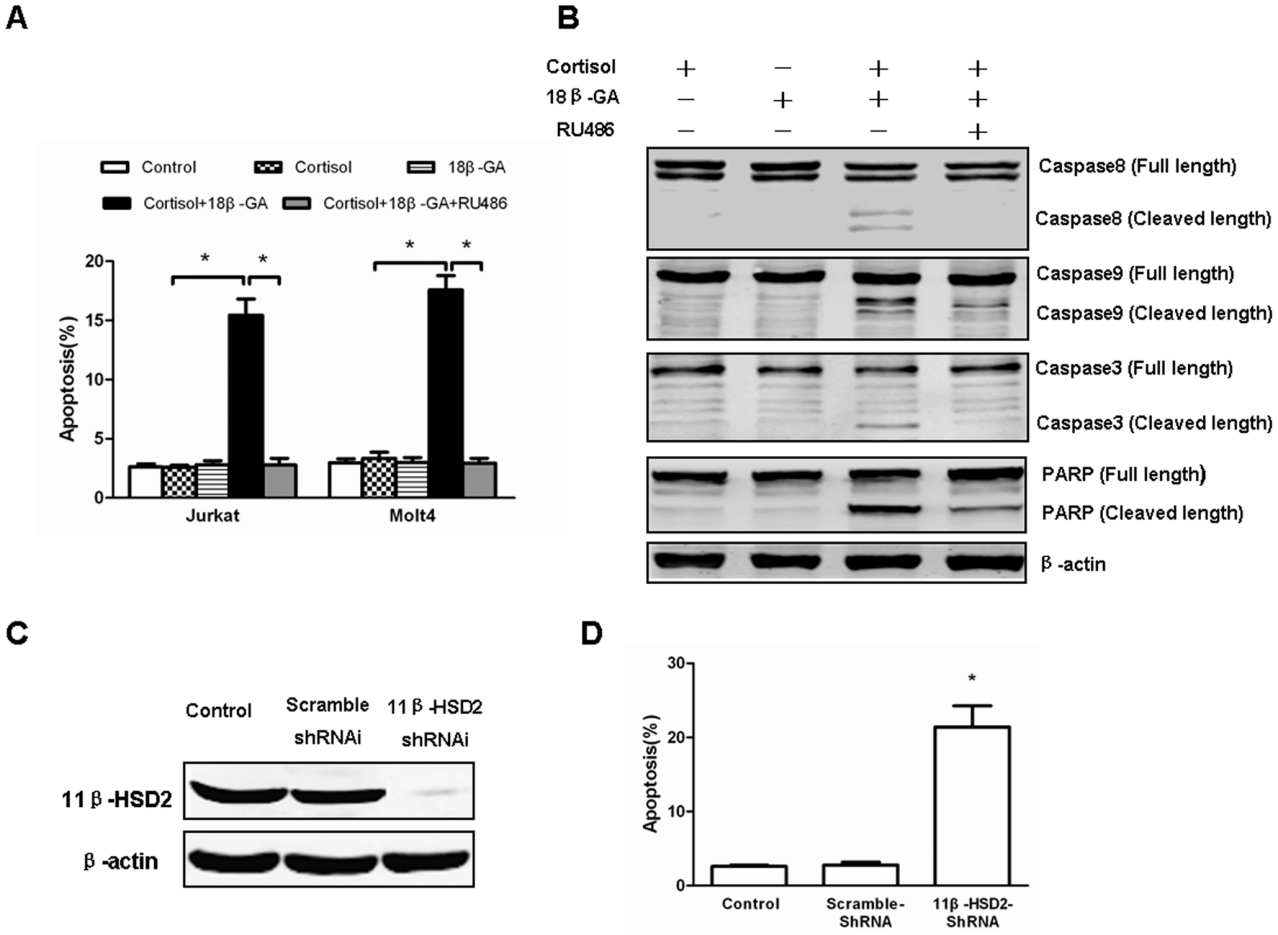
Inhibition of 11β-HSD2 activity enhances susceptibility to cortisol-induced apoptosis. (A) Jurkat and Molt4 cells were treated with 11β-HSD inhibitor 18β-GA (5 µM) plus 10 µg/ml cortisol in the presence or absence of GC receptor antagonist RU486 (1 µM) for 48 hours. Cells were then analyzed for apoptosis by flow cytometry using Annexin V/PI binding. Data are shown as mean±SD of 3 independent experiments. * indicates p<0.05. (B) Immunoblot analysis of Jurkat cells with different treatments as indicated was performed using anti–caspase-8, caspase-9, caspase-3 and PARP antibodies. The expression levels of β-actin in the lysates served as the loading control. (C) Western blots were used to examine 11β-HSD2 expression in 11β-HSD2 knockdown Jurkat cells. Wild-type (WT) and nonsense scramble shRNA (SCR)-transfected cells were used as controls. (D) WT, SCR-transfected and 11β-HSD2 knockdown Jurkat cells were treated with 10 µg/ml cortisol for 48 hours. Cells were then analyzed for apoptosis by flow cytometry using Annexin V/PI binding. Data are shown as mean±SD of 3 independent experiments. * p<0.05 *vs.* control cells.

To clarify whether the ectopic expression of the enzyme is responsible for the GC resistance, we examined the effect of 11β-HSD2 inhibition on cell apoptosis. Thus, Jurkat and Molt4 cells were treated with the specific 11β-HSD inhibitor 18β-GA and cortisol for 48 hours before analyzing cell apoptosis. We observed 18β-GA significantly increased cell Annexin V-binding, demonstrating that 18β-GA and cortisol acted synergetically in inducing cell apoptosis. Further, results demonstrated that RU486, a GR antagonist blocked apoptosis of both cells treated with 18β-GA and cortisol, implying the increased cell apoptosis was mediated by the ability of 18β-GA to enhance the proapoptotic effect of cortisol (Fig. 3A). Although 18β-GA inhibits both 11β-HSD1 and 11β-HSD2, the effect of 18β-GA is probably by the inhibition of 11β-HSD2 because 11β-HSD1 mRNA was only minimally expressed in both cells (data not shown) and only cortisol, a substrate for the enzyme, was present in the culture system. Additionally, western blot analysis showed activation of caspase-8, -9, -3 and cleaved PARP in cortisol-treated Jurkat cells after 11β-HSD2 inhibition, which is another evidence indicative of elevated cell apoptosis (Fig. 3B).

To further clarify the definite role of 11β-HSD2 in cell resistance to cortisol, specific shRNA (using the lentivirus shRNA expression vector system) was used to knockdown 11β-HSD2 in Jurkat cells. 11β-HSD2 protein expression was confirmed by Western blots (Fig. 3C). Similar to the effect of 18β-GA, 11β-HSD2 knockdown made Jurkat cells become highly sensitive to cortisol-induced apoptosis as compared to wild-type and scramble-transfected control cells (Fig. 3D), strongly suggesting that GC resistance in Jurkat cells was partly related to the presence of 11β-HSD2.

### Bortezomib Pretreatment Sensitizes Jurkat Cells to Cortisol-induced Apoptosis and G1 Cell Cycle Arrest

**Figure 4:**
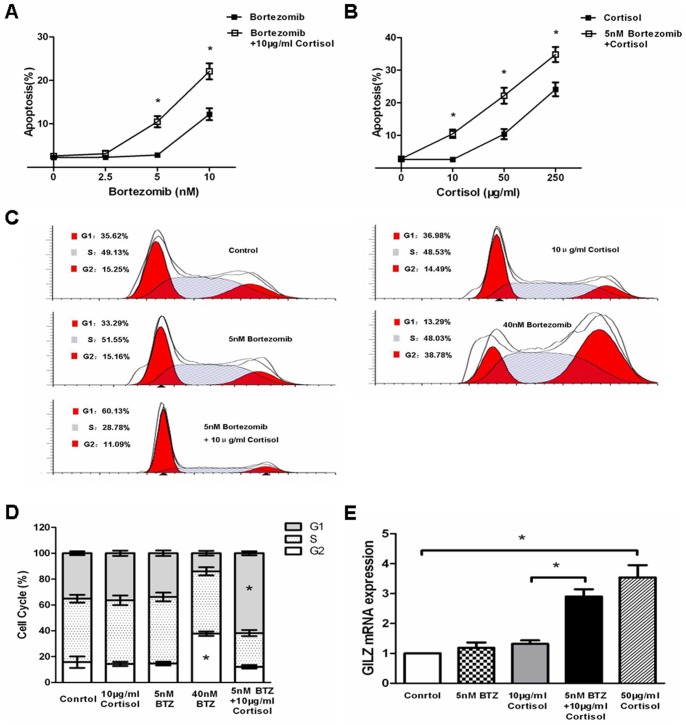
Pretreatment with bortezomib sensitizes Jurkat cells to cortisol-induced apoptosis and G1 cell cycle arrest. (A) Jurkat cells were pretreated with indicated concentrations of bortezomib (2.5–10 nM) for 24 hours. Cells were collected and washed once with medium. Afterwards, cells were treated with 10 µg/ml cortisol for 48 hours. Cells were then analyzed for apoptosis by flow cytometry using Annexin V/PI binding. Data are shown as mean±SD of triplicate samples. *p<0.05 *vs.* the cells treated with bortezomib alone. (B) Jurkat cells were pretreated with 5 nM bortezomib for 24 hours. Cells were collected and washed once with medium. Afterwards, cells were treated with indicated concentrations of cortisol for 48 hours. Cells were then analyzed for apoptosis by flow cytometry using Annexin V/PI binding. Data are shown as mean±SD of triplicate samples. *p<0.05 *vs.* cells without bortezomib pretreatment. (C) Jurkat cells were pretreated with 5 nM bortezomib (BTZ) for 24 hours. The cells were collected and washed once with medium. Afterwards, cells were treated with 10 µg/ml cortisol for 48 hours and analyzed for cell cycle by flow cytometry. (D) Graphical representation of the data displayed in panel C as mean±SD of 3 independent experiments. * in G2 phase indicates p<0.05 *vs.* control cells. * in G1 phase indicates p<0.05 *vs.* cells treated with cortisol alone. (E) Jurkat cells were pretreated with 5 nM bortezomib for 24 hours. The cells were collected and washed once with medium. Afterwards, cells were treated with 10 µg/ml cortisol for 48 hours and analyzed for GILZ mRNA expression by quantitative real-time PCR. Data are shown as mean±SD of triplicate samples. * indicates p<0.05.

Treatment of Jurkat cells with increasing doses of bortezomib resulted in direct cytotoxicty associated with enhanced apoptotsis (Fig. 4A). Different studies demonstrated that bortezomib potentiates the activity of other chemotherapeutics, in part by down-regulating chemoresistance pathways [13]. As shown in Fig. 1, Jurkat cells were resistant to cortisol-induced cytotoxicty. However, we observed that Jurkat cells underwent increased cortisol-induced apoptosis when pretreated with increasing doses of bortezomib (Fig. 4A). Particularly, pretreatment with low-dose bortezomib (5 nM) for 24 hours that did not exert any direct cytotoxicty significantly enhanced the potency of cortisol-induced apoptosis (Fig. 4B).

It is known that GC induces G1 cell cycle arrest in GC-induced T-cell apoptosis [16]. Whereas, bortezomib has been demonstrated to greatly arrest Jurkat cells at G2 phase in our study (Fig. 4C) and others [17]. We observed more cells were arrested by cortisol at G1 phase when pretreated with low-dose bortezomib (Fig. 4C and D), suggesting that bortezomib pretreatment may enhance the effects of cortisol on Jurkat cells.

GC-induced leucine zipper (GILZ), an important mediator of GC action, is up-regulated by GC [18]. Consistently, our results also showed that cortisol dose-dependently enhanced GILZ mRNA level in Jurkat cells (Fig. 4E). To further clarify the effects of botezomib pretreatment on cell response to cortisol, we investigated the level of GILZ mRNA in Jurkat cells. As expected, cortisol increased GILZ mRNA in bortezomib-pretreated Jurkat cells (Fig. 4E), confirming the role of low-dose bortezomib in sensitizing Jurkat cells to GC.

### Bortezomib Decreases 11β-HSD2 mRNA and Protein Expression as well as Enzyme Activity in a Dose-dependent Manner

**Figure 5:**
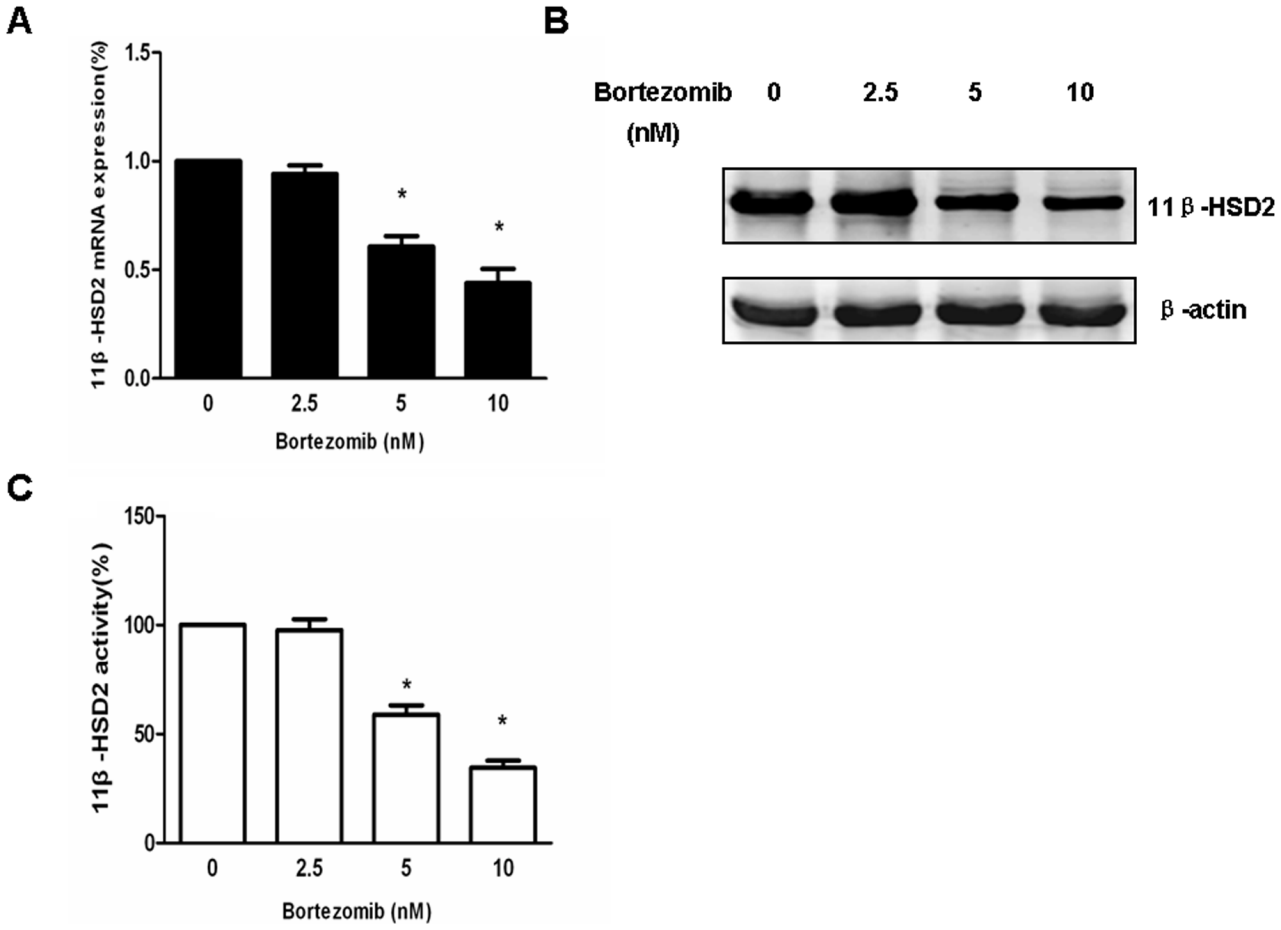
Bortezomib dose-dependently inhibits 11β-HSD2 mRNA and protein expression as well as enzyme activity. (A) Jurkat cells were exposed to the indicated concentrations of bortezomib for 12 hours and 11β-HSD2 mRNA levels were measured by quantitative real-time PCR and normalized to β-actin levels. Data are shown as mean±SD of triplicate samples. *p<0.05 *vs.* untreated cells. (B) Jurkat cells were exposed to the indicated concentrations of bortezomib for 24 hours and 11β-HSD2 protein levels were measured by immunoblot analysis using anti–11β-HSD2 antibody. The expression levels of β-actin in the lysates served as the loading control. One representative experiment is presented. (C) Jurkat cells were exposed to the indicated concentrations of bortezomib for 24 hours and 11β-HSD2 enzyme activities were determined by measuring production of cortisone from 100 nM cortisol added to the medium for 24 hours. The 11β-HSD2 activity was expressed as a percentage: pmol cortisone produced per 10^6^ cells of the treated cells/pmol cortisone produced per 10^6^ cells of the untreated cells. Data are shown as mean±SD of triplicate samples. *p<0.05 *vs.* untreated cells.

As mentioned above, high expression of 11β-HSD2 has been demonstrated in Jurkat cells and inhibition of 11β-HSD2 could restore cell susceptibility to cortisol. Therefore, we evaluated if bortezomib pretreatment improved GC cell sensitivity by regulating 11β-HSD2 expression. Jurkat cells were treated with increasing amounts of bortezomib (2.5–10 nM), and the mRNA expression levels of 11β-HSD2 were examined 12 hours posttreatment. As shown in Fig. 5A, 11β-HSD2 levels in Jukat cells were significantly reduced by 5 nM bortezomib and further decreased in a dose-dependent manner. The control of 11β-HSD2 by bortezomib was then examined by quantifying 11β-HSD2 protein levels and enzyme activity 24 hours posttreatment using immunoblotting and enzyme activity assay respectively. Consistent with the reduced mRNA levels, bortezomib also dose-dependently resulted in inhibition of both 11β-HSD2 protein level and cotisol-cortisone converting activity (Fig. 5B and C), indicating 11β-HSD2 was exactly a downstream target of bortezomib action.

### Bortezomib Decreases 11β-HSD2 Expression through p38 MAPK Signaling Pathway

**Figure 6:**
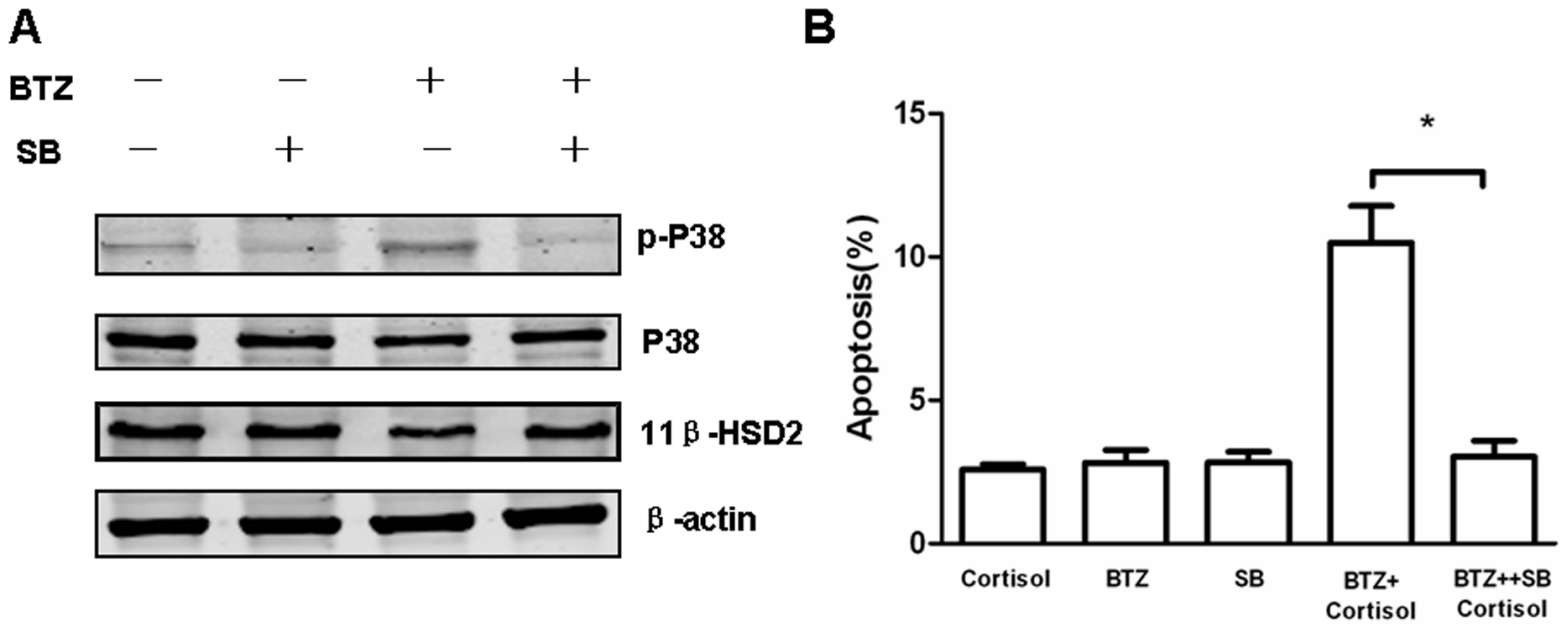
The effects of the p38 MAPK inhibitor on increased sensitivity to cortisol in Jurkat cells pretreated with bortezomib. (A) Jurkat cells were pretreated with 5 nM bortezomib (BTZ) in the presence or absence of 10 µM SB203580 (SB), a selective p38 inhibitor for 24 hours. Immunoblot analysis of Jurkat cells after different treatments as indicated in the figure was probed with anti-p-p38 MAPK, anti-p38 MAPK and anti-11β-HSD2, to determine the activation of p38 MAPK and the expression of 11β-HSD2. The expression levels of β-actin in the lysates served as the loading control. (B) Jurkat cells were pretreated with 5 nM bortezomib (BTZ) in the presence or absence of 10 µM SB203580 (SB) for 24 hours. Cells were collected and washed once with medium. Afterwards, cells were treated with 10 µg/ml cortisol for 48 hours. Cells were then analyzed for apoptosis by flow cytometry using Annexin V/PI binding. Data are shown as mean±SD of triplicate samples. *p<0.05 *vs.* cortisol-treated cells pretreated with bortezomib in the absence of SB203580.

It has been reported that the activation of p38 MAPK enhances GC sensitivity in GC-resistant T lymphoblastic cells [16]. Bortezomib has been demonstrated to exert numerous biological effects that include activating p38 MAPK [19]. Therefore, we hypothesize if bortezomib regulates 11β-HSD2 expression through p38 MAPK signaling pathway. Jurkat cells were treated with bortezomib in the presence or absence of SB203580, a selective p38 inhibitor, to examine 11β-HSD2 expression. Immunoblotting showed low-dose bortezomib resulted in phosphorylation of p38 MAPK while decreasing 11β-HSD2 (Fig. 6A). Furthermore, co-treatment of SB203580 reversed bortezomib-induced inhibition of 11β-HSD2 (Fig. 6A), indicating p38 MAPK may play an important role in decreasing 11β-HSD2 by bortezomib. As expected, we further demonstrated p38 inhibitor SB203580 reversed cortisol-induced apoptosis in Jurkat cell pretreated with bortezomib (Fig. 6B).

## Discussion

GC action is reported to be dependent on 11 beta-HSD activities contained in tissues, the amount of GC receptors in target cells as well as the concentration of circulating GC [20]. Many studies owe GC resistance to the GR within unresponsive cells. A deficient up-regulation of the GR and its downstream target, GC-induced leucine zipper (GILZ) was found in GC-resistant subclones generated from a GC-sensitive cell line, which was attributed to mutational events [3]. Conversely, Beesley et al. examined 15 T-ALL cell lines and showed that GR resistance could not be explained by mutations in GR or variations in levels of its expression [4]. Additionally, the GR protein expression showed no difference between GR-responsive and GR-resistant patients in a case-control study [5].

Our data showed the basal level of GRα mRNA was lower in Jurkat and Molt4 cells compared to Daudi and Raji cells. Treatment with cortisol resulted in no auto-induction of GRα in Jurkat and Molt4 cells thus giving an approximately 8-fold difference in GRα mRNA between Jurkat and Daudi cells. However, even a 10-fold higher dose of cortisol could not induce apoptosis in Jurkat cells to the levels achieved in Daudi cells, suggesting other mechanisms apart from the low numbers of GR were involved in GC resistance. Jurkat cells have been demonstrated to express one wild-type and one mutated (R477H) GR allele. However, GC-sensitive cells transduced with the same mutated (R477H) GR underwent GC-induced apoptosis to the same degree and with similar kinetics as those transduced with wide type GR, thus indicating the mutation alone did not sufficiently account for the GC-resistant phenotype of Jurkat cells [21]. Here we have shown that high levels of 11β-HSD2 are present in Jurkat cells and inhibition of 11β-HSD2 by 18β-GA sensitizes Jurkat cells to GC. Similar results were also observed in Molt4 cells consistent with findings from Sai et al [2]. We further observed that 11β-HSD2 knockdown by specific shRNA sensitized Jurkat cells to cortisol-induced apoptosis, confirming 11β-HSD2 contribution to GC resistance in Jurkat cells. However, we demonstrated there was no 11β-HSD2 expression in normal peripheral blood T cells, in accord with pervious findings of 11β-HSD2 absent in normal tissues but present in malignant counterparts [10], [22]. Moreover, Rabbitt et al. reported that cells transfected with cDNA encoding 11β-HSD2 significantly increased cell proliferation [23], which may explain the possible role 11β-HSD2 played in the formation of T-lymphoblastic leukemias. Although no 11β-HSD2 expression was found in B-NHL Daudi and Raji cells, maybe it is because T cells developed in a way different from B cells. As is known, T cells undergo positive and negative selection in thymus before they mature and transfer to the periphery. Specifically, it is thought that negative selection induces the death of thymocytes that bind strongly to self-peptide [24]. It has long been known that the apoptosis in thymocytes could be induced by GC, whose levels are able to be controlled within lymphoid organs through oxidative inactivation by 11β-HSD2 [20]. It is possible that the presence of 11β-HSD2 makes these T-lymphoblastic leukemic cells escape negative selection resulted from GC-induced apoptosis, leaving them to survive and finally transform into lymphoblastic cells. It has been shown that caspase- 8, -9 and -3 activity is essential for GC-induced apoptosis in thymocytes as well as T-ALL cells [25]. Consistently, we observed processing of caspase-8,-9 and -3 and cleaved PARP in cortisol-treated Jurkat cells after 11β-HSD2 inhibition, indicating that both death receptor–dependent (caspase 8) and –independent (caspase 9) apoptotic pathways were involved.

Bortezomib, a new selective proteasome inhibitor drug, not only directly induces apoptosis in cancer cells, but also sensitizes these cells to chemotherapy [13], [26], [27]. In our study, we demonstrated that bortezomib also possessed the ability to enhance cell sensitivity to cortisol even at a non-cytotoxic low dose (5 nM). The improved sensitivity to cortisol by bortezomib pretreatment could be confirmed in 3 aspects. First, elevated Annexin V-binding indicated elevated cellular apoptosis in cortisol-treated Jurkat cells following bortezomib pretreatment. Second, cortisol arrested increased cells at G1 stage after bortezomib intervention, which is characteristic of enhanced cortisol effects considering cells were arrested at G2 stage with bortezomib treatment alone. Third, GILZ has a distinguished reputation as a critical mediator of GC effects and also participates in the regulation of cell apoptosis by GC [28]. Therefore, up-regulated GILZ mRNA is another solid evidence for the definite effects of bortezomib on sensitizing Jurkat cells to cortisol action. Although the same effects need to be confirmed by further studies in animal models which we plan to conduct in the near further, the in vitro results strongly indicate the synergistic advantage of combining proteasome inhibitor with cortisol clinically.

The mechanism by which bortezomib affects cell sensitivity to cortisol is elusive. It is well known that most GC actions are mediated by GR. Methotrexate has been reported to be a steroid sparing agent by means of increasing the expression of the active receptor (GRα) and/or decreasing the expression of the dominant negative receptor (GRβ) [29]. Whereas, we observed neither the number of GR nor the ratio of GRα to GRβ was changed in Jurkat cells after 5 nM bortezomib treatment (data not shown). Taking into account the potential role 11β-HSD2 plays in GC resistance, further we testified whether bortezomib had any effects on 11β-HSD2, a cortisol-inactivating enzyme in Jurkat cells. Interestingly, bortezomib decreased 11β-HSD2 mRNA and protein expression as well as enzyme activity in a dose-dependent manner, which could explain its effects in improving GC susceptibility. Further, the ability of low dose (5 nM) bortezomib to inhibit 11β-HSD2 activity and enhance GC sensitivity prompted us to clarify the intracellular mechanism. It has been demonstrated that bortezomib exerts numerous biological effects through NF-κB, AKT or MAPKs signal pathways [30], [31]. In this study, we observed 5 nM bortezomib did not have any direct cytotoxic effects and NF-κB inhibitory activity (data not shown), which was in line with findings from Kashkar el al. [13]. Numerous reports have indicated that 11β-HSD2 can be regulated by MAPKs signalling [32], [33]. Furthermore, MAPKs have been proven to modify GC sensitivity, namely that the activation of extracellular signal-regulated protein kinase (ERK) and p38 MAPK reduce and enhance GC sensitivity, respectively [16]. We showed that blockade of p38 MAPK using SB203580, a selective p38 inhibitor abrogated the effects of bortezomib on inhibiting 11β-HSD2 and failed to sensitize cell to cortisol-induced apoptosis, implying p38 MAPK pathway activation could be an excellent potential therapeutic strategy in overcoming GC resistance.

Although it has been reported that non-metabolisable GC dexamethasone is poorly inactivated by 11β-HSD2 [2], possibly accounting for why dexamethasone is widely applied in clinical fields, cortisol is still superior to dexamethasone in some respects. For example, cortisol has a shorter half-life of 8 hours compared to dexamethasone of 54 hours, which can reduce the adverse effects and complications of GC such as immunodeficiency, hyperglycemia and steroid-induced osteoporosis [34]. In addition, GCs also represent the mainstay strategy for diseases caused by an overactive immune system, such as allergies, asthma, autoimmune diseases and sepsis, which are suitable for being treated by short-acting cortisol.

Taken together, here we demonstrate at least two mechanisms responsible for GC resistance in leukemia T cells: failure of GR auto-induction and presence of cortisol-inactivating 11β-HSD2 enzyme. We also show that upon bortezomib pretreatment, Jurkat cells exhibit an increased sensitivity to cortisol, suggesting proteasome inhibitors could be potential GC sparing agents, specifically for diseases with GC resistance resulted from 11β-HSD2 presence.
